# Association of Serum IL-17 and IL-23 Cytokines With Disease Activity and Various Parameters of Rheumatoid Arthritis in Indian Patients

**DOI:** 10.7759/cureus.49654

**Published:** 2023-11-29

**Authors:** Jhasaketan Meher, Suprava Patel, Rachita Nanda, Md Sabah Siddiqui

**Affiliations:** 1 General Medicine, All India Institute of Medical Sciences, Raipur, Raipur, IND; 2 Biochemistry, All India Institute of Medical Sciences, Raipur, Raipur, IND

**Keywords:** il-23, il-17, functional outcome, early/advanced ra, disease activity, cytokine, rheumatoid arthritis

## Abstract

Introduction

Interleukin-23/T helper 17 (IL-23/Th17) axis cytokine has been thought to be a critical pathway for rheumatoid arthritis (RA) disease development and its association with disease severity, joint erosion, and functional outcome. There is a paucity of data on the role of IL-23/Th17 axis cytokines in an Indian RA subset of patients. We aimed to determine the association between serum cytokines (interleukin-17 [IL-17] and [IL-23]) and disease activity as well as with clinical and biochemical parameters of RA patients.

Methods

In this observational cross-sectional study, 84 consecutive RA cases were recruited after obtaining consent. Serum IL-17 and IL-23 levels were measured by the enzyme-linked immunosorbent assay (ELISA) method. Clinical and laboratory parameters, disease activity score 28-erythocyte sedimentation rate (DAS28-ESR), and Health Assessment Questionnaire-II (HAQ-II) were recorded. Correlation of cytokines with various clinical and biochemical parameters was elicited.

Results

Only C-reactive protein (CRP) correlated positively with IL-23 (rs = 0.26, p = 0.014) but not the ESR. Both IL-17 and IL-23 levels showed an insignificant, weak positive correlation with the disease activity DAS28 (rs = 0.18, p = 0.097; rs = 0.12, p = 0.259, respectively). Neither IL-17 nor IL-23 levels differed among the disease severity group (p = 0.13, p = 0.215). Only the IL-23 level positively correlated with functional status (HAQ-II) (rs = 0.28, p = 0.009). IL-17 level was higher in advanced RA as compared to early RA (p = 0.028). Both IL-17 and IL-23 levels did not vary within the different subgroups (age, obesity, disease-modifying drugs/steroid/biologics use, and serology status).

Conclusion

Females had higher IL-23 levels than males. Advanced RA had higher IL-17 levels than early RA. The cytokine levels were not influenced by factors like age, duration of disease, serology status, or drugs. Neither of the cytokines correlated significantly with disease severity. Higher IL-17 levels may have a role in the progression of early non-erosive to chronic erosive arthritis. Higher IL-23 levels may signal a bad functional outcome.

## Introduction

Rheumatoid arthritis (RA) is an autoimmune disorder characterized by bilateral symmetric polyarthritis, leading to joint erosions and joint destruction in chronic untreated cases [1]. The worldwide prevalence of RA is approximately 0.5%-1% among adults, while it ranges from 0.28% to 0.7% in India [2].

Several cytokines have been implicated in the pathogenesis of RA as they upregulate the inflammatory cascade, resulting in synovial proliferation, osteoclastic bone resorption, and joint erosion. T cells, particularly T helper 1 cells (Th1) and their associated cytokines, play a crucial role in the initiation and progression of RA. More recently, T helper 17 (Th17) cells and their cytokine family, including interleukin-23 (IL-23) and interleukin-17 (IL-17), have gained attention as a critical pathway in disease development. They are also associated with disease duration, disease severity, joint erosion, and functional outcomes [3].

However, there is a lack of data regarding the role of Th17/IL-23/IL-17 axis cytokines in the Indian subset of patients and their associations with various disease factors. Therefore, this study was conducted to investigate any potential associations between these cytokines and clinical and biochemical parameters, such as disease activity, disease duration, serology status, and functional status.

## Materials and methods

Study setting

This cross-sectional observational study was conducted at the All India Institute of Medical Sciences (AIIMS) in Raipur, India. Cases were recruited from the General Medicine outpatient and inpatient departments between August 2021 and March 2022.

Objectives

The primary objective of this study was to investigate the association between serum cytokines (IL-17 and IL-23) and disease activity score 28 (DAS28). The secondary objective was to explore potential associations between cytokines and various clinical and biochemical parameters of the disease, such as age, gender, disease duration, serology status, medications, and functional status.

Inclusion and exclusion criteria

Participants aged above 18 years with either newly diagnosed or previously diagnosed RA were included. Pregnant or lactating women, RA patients with other overlapping inflammatory arthritis or connective tissue diseases, those with autoimmune inflammatory conditions other than RA, people with active infections, and individuals with psoriasis were excluded.

Methods

A total of 84 consecutive RA cases were included in the study after meeting the 2010 American College of Rheumatology (ACR)/European League Against Rheumatism (EULAR) criteria for RA classification. Consent was obtained from all eligible participants after confirming that they met the inclusion and exclusion criteria. Detailed medical histories were collected, and a comprehensive clinical examination was performed for each participant, including the documentation of all demographic data and clinical characteristics. Data on laboratory parameters and treatment regimens were also recorded. IL-17 and IL-23 levels were measured using the enzyme-linked immunosorbent assay (ELISA) method. Disease activity was assessed using the DAS28-erythrocyte sedimentation rate (DAS28-ESR) with the RheumaHelper app v4.2. Disease activity was categorized as remission (<2.6), mild (2.6-3.2), moderate (3.2-5.1), and severe (>5.1). Functional status was determined using the Health Assessment Questionnaire-II (HAQ-II). All data were electronically stored in EpiData software v3.1 (EpiData Association, Denmark), and data analysis was conducted using the Statistical Package for the Social Sciences (SPSS) software v21 (IBM Corp., Armonk, NY). The study adhered to the International Council for Harmonization of Technical Requirements for Pharmaceuticals for Human Use (ICH) guidelines for good clinical practice, the Helsinki Declaration, and applicable standard operating procedures. The study received approval from the institute's ethics committee at AIIMS, Raipur, India.

Statistical analysis

Data from participants and laboratory results were entered into EpiData v3.1 software and analyzed using SPSS software v21. Quantitative data were presented as means and standard deviations (SD) or medians with interquartile ranges (IQR, Q1-Q3), while qualitative data were presented as percentages or proportions. The Pearson correlation test was employed to determine the relationship between serum cytokines and disease activity scores and functional status if the data followed a normal distribution. For non-normally distributed data, the Spearman correlation test was used. The association between categorical variables was examined using the Chi-square test. The correlation between categorical and ordinal variables was assessed with the Kruskal-Wallis test if the data did not follow a normal distribution. A p-value < 0.05 was considered statistically significant, and multivariate regression analysis was conducted to identify independent associations in the presence of confounding factors.

## Results

The study revealed a higher prevalence of females than males (n = 76, 90.5%, and n = 8, 9.5%, respectively). In this study, most cases (n = 74, 88.1%) were in the age range of 18-60 years, with a median age of 44.73 ± 11.04 years. However, elderly RA (>60 years) comprised 11.9% of the cases. The median duration of morning stiffness was 60 minutes (16.5-120). About 75% (n = 63) of cases had a disease duration of more than one year. The overall median disease duration was 36 months (IQR: 13.5-69). Only a few cases had extraarticular system involvement, including uveitis in one case, digital gangrene in two cases, and interstitial lung disease in one case.

Most of the cases were already taking conventional disease-modifying drugs (cDMARDs) before being recruited for the study. Methotrexate, hydroxychloroquine, sulfasalazine, and leflunomide were taken by 90.5% (n = 76), 67.9% (n = 57), 33.3% (n = 28), and 2.4% (n = 2) of cases, respectively. Steroid use (prednisolone and methylprednisolone) was found in 48.8% of cases (n = 41). Only four cases (4.8%) had received biologics (rituximab) when cDMARDs failed or when there were extraarticular manifestations like interstitial lung disease (ILD), vasculitis, and uveitis. Most of the cases had a normal body mass index (BMI). However, 9.52% of cases were obese, while 16.66% of cases were overweight. The mean BMI was 22.79 ± 4.97 kg/m^2^. The mean DAS28-ESR was 5.22 ± 1.68. The majority of cases (57.14%, n = 48) had high disease activity, while 25% (n = 21) and 11.9% (n = 10) had moderate and mild disease activity, respectively. Only 6% (n = 5) of cases were in remission. The baseline characteristics of all cases are described in Table 1.

**Table 1:** Baseline characteristic of demographic, clinical, and laboratory parameters SD: Standard deviation; IQR: Interquartile range; RA: Rheumatoid arthritis; cDMARDs: Conventional disease-modifying antirheumatic drugs; BMI: Body mass index; DAS28: Disease activity score 28; ESR: Erythrocyte sedimentation rate; CRP: C-reactive protein; IL-23: Interleukin-23; IL-17: Interleukin-17; HAQ-II: Health Assessment Questionnaire-II.

Characteristics	Frequency: mean ± SD or median (IQR)
Age (years)	44.73 ± 11.04
Male:female (n = 84)	8:76 (90.5% vs 9.5%)
Disease duration (in months)	36 (13.5-69)
Morning stiffness (in minutes)	60 (16.5-120)
Deformity (n = 84)	17 (20.2%)
Family history of RA (n = 84)	11 (13.1%)
cDMARD use (n = 84)	67 (79.8%)
Steroid use	41 (48.8%)
BMI (kg/m^2^)	22.79 ± 4.97
Swollen joint count	3.45 ± 4.43
Tender joint count	8.06 ± 7.92
DAS28	5.22 ± 1.68
ESR (mm in 1^st^ hour) (n = 84)	61.5 (38.5-98)
CRP (mg/dl) (n = 83)	11.7 (2.22-23.28)
IL-23 (pg/ml) (n = 84)	981.87 (793.25-1205.0)
IL-17 (pg/ml) (n = 84)	103.72 (39.91-524.13)
HAQ-II	12.34 ± 7.35

The median values of rheumatoid factor (RF) (n = 67) and anti-cyclic citrullinated peptide antibody (anti-CCP) (n = 83) were 88.0 IU/ml (15.0-120.0) and 242.3 RU/ml (38.06-251.0), respectively. The median values of IL-17 (n = 84) and IL-23 (n = 84) were 103.72 pg/ml (39.91-524.13) and 981.87 pg/ml (793.25-1205.0), respectively. IL-17 showed no correlation with RF and anti-CCP (rs = 0.147, p = 0.236; rs = -0.015, p = 0.896). Similarly, IL-23 did not show any correlation with RF and CCP (rs = 0.047, p = 0.707; rs = 0.063, p = 0.571). The functional status, as measured by HAQ-II, was 12.34 ± 7.35.

**Figure 1:**
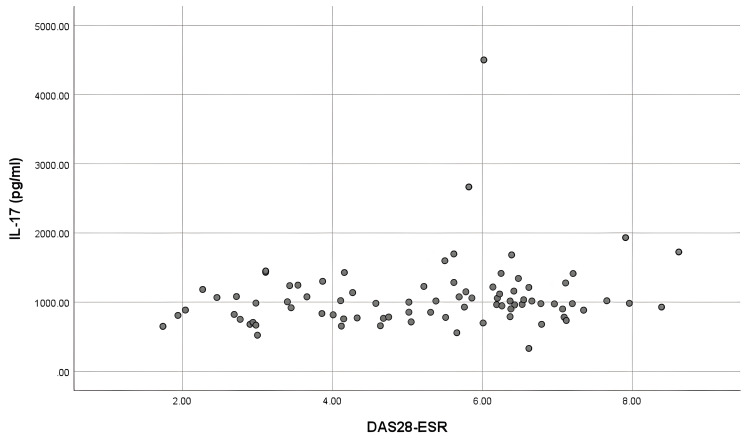
Correlation of serum IL-17 with disease activity (DAS28-ESR) IL-17: Interleukin-17; DAS28-ESR: Disease activity score 28-erythrocyte sedimentation rate.

**Figure 2:**
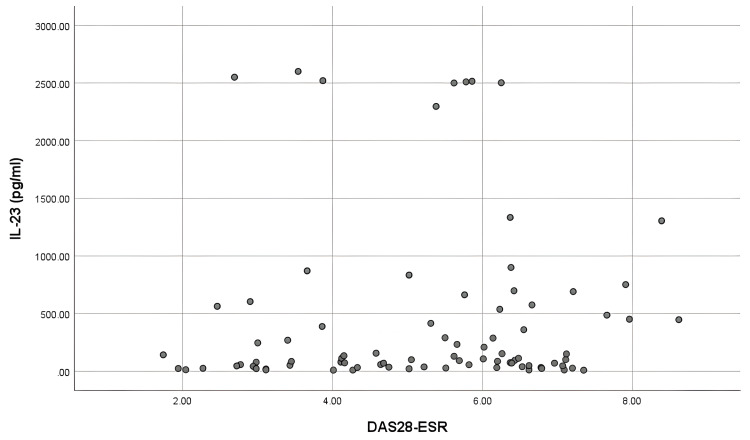
Correlation of serum IL-23 with disease activity (DAS28-ESR) IL-23: Interleukin-17; DAS28: Disease activity score 28-erythrocyte sedimentation rate.

The IL-17 and IL-23 levels did not show any correlation with the disease activity (rs = 0.183, p = 0.097; rs = 0.125, p = 0.259), respectively (Figures 1, 2).

**Figure 3:**
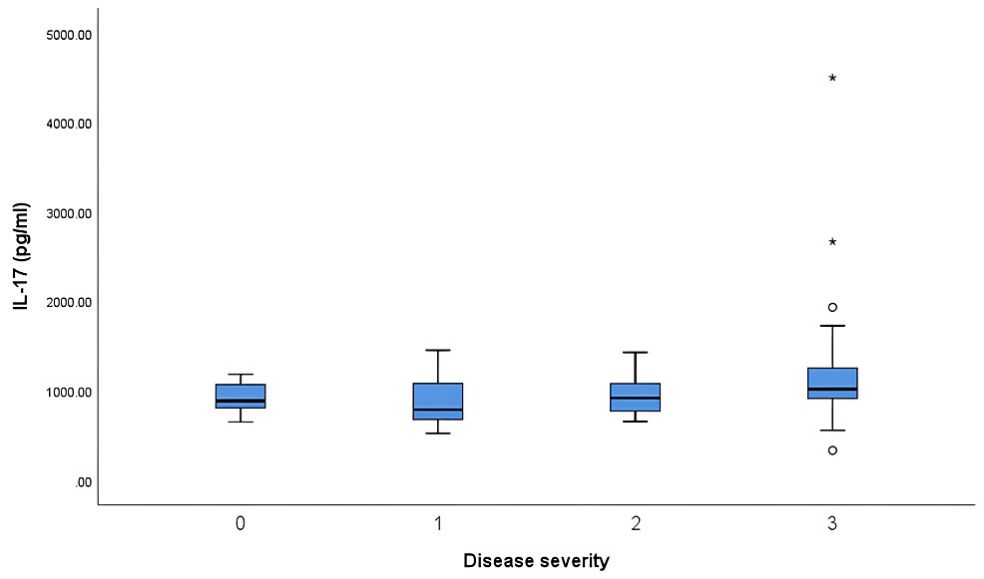
Correlation of serum IL-17 with different disease severity categories (DAS28) IL-17: Interleukin-17; DAS28: Disease activity score 28. 0 = Remission, 1 = Mild, 2 = Moderate, 3 = Severe. * Extreme outlier (data beyond three interquartile ranges from the median). º Mild outlier (data located beyond 1.5 interquartile range from the median).

**Figure 4:**
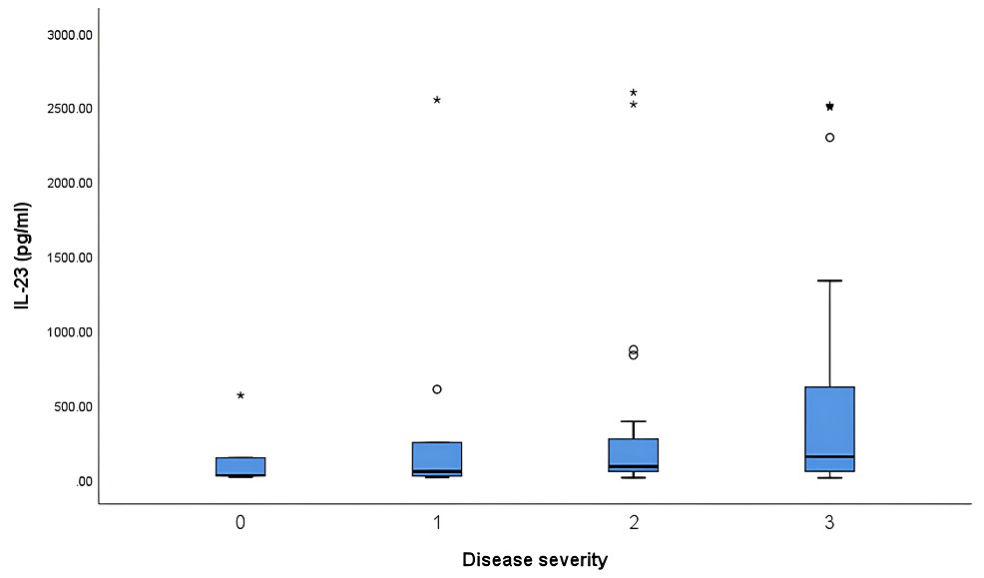
Correlation of serum IL-23 with different disease severity categories (DAS28) IL-23: Interleukin-23; DAS28: Disease activity score 28. 0 = Remission, 1 = Mild, 2 = Moderate, 3 = Severe. * Extreme outlier (data beyond three interquartile ranges from the median). º Mild outlier (data located beyond 1.5 interquartile range from the median).

Upon further analysis, it was found that there was no difference in the IL-17 and IL-23 levels across the different disease severity categories (Table 2 and Figures 3, 4).

**Table 2:** Correlation between cytokines and disease severity category The Kruskal-Wallis test is significant when the p-value (p) is less than 0.05. IL-17: Interleukin-17; IL-23: Interleukin-23.

N = cases	Variables		Result
84	IL-17	Versus disease severity category	p = 0.130
84	IL-23		p = 0.215

It was also noted that IL-17 had a weak positive correlation with IL-23, which was statistically significant (rs = 0.221, p = 0.044). Among the cytokines (IL-17, IL-23), only IL-23 revealed a statistically significant weak positive correlation with functional status (HAQ-II) (rs = 0.284, p = 0.009). In the subgroup analysis, both IL-17 and IL-23 were compared in different subgroups, such as younger versus elderly RA, male versus female, early versus advanced RA, seropositive versus seronegative, cDMARDs/steroid versus naive, etc. It was found that serum IL-17 levels were higher in the advanced disease group compared to the early disease group. Also, female patients had higher serum IL-23 levels than male patients (Table 3).

**Table 3:** Comparison of cytokines in different subgroups A Chi-square test with a p-value (p) of less than 0.05 is considered significant. IL-17: Interleukin-17; IL-23: Interleukin-23; RA: Rheumatoid arthritis; cDMARDs: Conventional disease-modifying antirheumatic drugs; RF: Rheumatoid factor; CCP: Cyclic citrullinated peptide.

Comparison group	IL-17	IL-23
Male vs female	p = 0.710	p = 0.009
Younger vs elderly	p = 0.755	p = 0.350
Nonobese vs obese	p = 0.804	p = 0.804
Early vs advanced RA	p = 0.028	p = 0.223
Deformity vs no-deformity	p = 0.277	p = 1.000
cDMARDs vs naive	p = 1.000	p = 0.277
Steroid vs naive	p = 1.000	p = 0.175
Biologic vs naive	p = 0.474	p = 0.474
RF+ve vs RF-ve	p = 0.723	p = 0.723
CCP+ve vs CCP-ve	p = 0.549	p = 0.956

Many confounders that might have been associated with the outcome of the dependent variables (IL-17 and IL-23) were considered. Multiple linear regression was used to test the influence of independent variables like age, gender, duration of disease, use of DMARDs, steroids, DAS28, RF, and anti-CCP on cytokine levels (IL-17 and IL-23). The overall regression was insignificant for both IL-17 and IL-23 (R2 = 0.131, p = 0.381; R2 = 0.085, p = 0.709). It was also found that none of the factors were independently associated with either IL-17 or IL-23 (Table 4).

**Table 4:** The regression table presents the relationship between cytokines and disease activity, along with the factors that influence disease activity. p-value (p) less than 0.05 is considered statistically significant. IL-17: Interleukin-17; IL-23: Interleukin-23; cDMARDs: Conventional disease-modifying antirheumatic drugs; ESR: Erythrocyte sedimentation rate; DAS28: Disease activity score 28; HAQ-II: Health Assessment Questionnaire-II.

Independent variables	Dependent variables			
	IL-17		IL-23	
	β	p-value	β	p-value
Age	-7.864	0.154	3.280	0.687
Gender	66.294	0.743	-302.884	0.315
Disease duration	.553	0.491	.103	0.931
cDMARDs	-82.723	0.681	140.122	0.639
Methotrexate use	-73.028	0.550	79.742	0.661
Steroid use	127.519	0.311	162.773	0.384
ESR	-1.521	0.425	.163	0.954
DAS28	33.752	0.496	-6.711	0.927
HAQ-II	11.272	0.220	3.862	0.776

## Discussion

IL-23 belongs to the interleukin 12 (IL-12) cytokine family and is secreted by activated macrophages. Elevated IL-23 levels promote the differentiation of naive CD4+ T cells into T helper 17 cells (Th17), resulting in the secretion of tumor necrosis factor-alpha (TNF-α), interleukin-1 beta (IL-1β), interleukin-21 (IL-21), and interleukin-17 (IL-17) from Th17 cells as well as interleukin-6 (IL-6) from macrophages and dendritic cells. IL-23Rα interacts with Janus kinase 2 (JAK2), leading to a signal transducer and activator of transcription 3 (STAT3) phosphorylation, which facilitates the development of Th17 cells [4]. IL-17 is implicated in both early and established RA disease, promoting the activation of fibroblast-like synoviocytes (FLS), osteoclastogenesis, and the recruitment and activation of neutrophils, macrophages, and B lymphocytes [5]. The differentiation of osteoclasts is significantly induced in the presence of IL-17, either directly or indirectly, through the upregulation of receptor activator of nuclear factor kappa-B ligand (RANKL) [6]. Higher levels of Th17 cells are observed in RA synovium compared to osteoarthritis [7]. Low, persistent levels may lead to the transformation of early arthritis into chronic, persistent arthritis [6]. The role of Th17 cytokines in disease pathogenesis and their correlation with different components of the disease requires extensive elaboration and debate. In this study, we elucidate the association of cytokines with various disease factors.

Age, gender, and cytokines

In our study, only IL-23 exhibited a significant difference between females and males, with higher levels in females (p = 0.009), whereas IL-17 did not exhibit any difference (p = 0.710). In this study, elderly RA patients (>60 years) comprised 11.9% of cases. Neither IL-17 levels nor IL-23 levels showed any difference between the younger and elderly RA groups (p = 0.755; p = 0.350).

Early RA, advanced RA, and cytokines

The association of cytokines with disease duration varies widely. IL-17 receptor signaling activation may serve as the trigger for the transformation of acute synovitis into chronic, persistent arthritis [8]. Early inflammatory arthritis may exhibit a different cytokine profile (IL-2, IL-4, IL-13, IL-17, IL-15, and fibroblast growth factor) in synovial fluid, which is typically not found in advanced diseases [9]. Animal studies have demonstrated the role of IL-17A in early RA, working in parallel with tumor necrosis factor (TNF). Blocking IL-17 is shown to decelerate the progression to chronic inflammatory arthritis and advanced disease [10]. In another study, it was found that the plasma level of IL-23 was higher in early RA compared to chronic RA and healthy volunteers, but the plasma level of IL-17A did not differ [11]. The majority of our cases had a longer duration of disease at presentation.

Our study showed a statistically significant higher level of IL-17 in advanced disease compared to early RA (p = 0.028), but the IL-23 level did not exhibit any difference between early versus late RA (p = 0.223). As the results are conflicting, further research is needed to support or refute the role.

Erosive RA, deformity, and cytokines

There has been debate regarding the role of cytokines in the development and progression of erosive arthritis in RA. IL-17 induces RANKL, which activates osteoclastic activity, resulting in joint erosion [4,5]. Published data indicate that IL-17 plays a significant role in early RA pathogenesis and subsequent erosive arthritis [10]. In a study, it was revealed that changes in IL-23 plasma levels from baseline to one year correlated with changes in disease severity and joint erosion (total sharp score) in two years as well as radiological deterioration [12]. Complete prevention of arthritis was demonstrated in mice deficient in IL-17 along with IL-1ra deficiency. It also highlighted the importance of developing erosive arthritis in IL-1 receptor-deficient mice [13]. IL-23 has been implicated in the pathogenesis of erosive arthritis in RA, with serum IL-23 levels directly correlating with radiological severity grading in RA patients [14]. In this study, neither of the cytokines (IL-17 and IL-23) exhibited any difference between deformity and non-deformity RA (p = 0.277 and p = 1.000, respectively).

Autoantibodies and cytokines

Limited studies have compared serology status (RF positive [RF+ve] or RF negative [RF-ve] and anti-CCP positive [CCP+ve] or anti-CCP negative [CCP-ve]) with Th17 cytokines. In one study, RF-ve RA patients had statistically insignificant higher mean serum IL-17A levels than RF+ve patients (12 ± 9.86 pg/mL and 10.82 ± 9.81 pg/mL, respectively) [15]. In another study, IL-23 levels had a significant correlation with RF (r = 0.48, p = 0.002) and anti-CCP antibodies (r = 0.35, p = 0.04) [16]. We did not find any correlation between cytokines and autoantibodies. However, in the subgroup analysis, none (RF+ve versus RF-ve and CCP+ve versus CCP -ve) revealed any significant difference in IL-17 or IL-23 levels within the subgroup.

cDMARDs/steroids and cytokines

The development of cDMARDs has changed the way we manage RA. Early use of cDMARDs has been shown to decrease erosions and deformity [17]. Most of our participants were already on cDMARDs before recruitment, with methotrexate being the most commonly used, followed by hydroxychloroquine, sulfasalazine, and leflunomide. Additionally, 48.8% of cases were on low- to moderate-dose steroids for a few weeks to months. Biologics such as TNF inhibitors, rituximab, tocilizumab, and synthetic biosimilars have been used to treat difficult-to-treat RA patients or in cases of cDMARD failure or extraarticular severe disease manifestations [18]. In our study, 4.8% of cases had received rituximab. The effect of cDMARDs, steroids, and biologics on changes in cytokine levels remains a topic of research.

Kageyama et al. conducted a comparative study to investigate the effect of infliximab versus cDMARDs plus placebo on serum IL-17 and IL-23 levels and found that IL-23 levels decreased in the infliximab group. However, the same was not observed in the methotrexate + placebo group at 14 and 30 weeks, and changes in IL-17 levels were not evident. At baseline, there was a positive correlation between IL-23 and DAS28 [19]. In a study by Lee et al., remission was more common in the low serum IL-17A group than in the high serum IL-17A group (47.6% versus 17.4%, p = 0.032) in tocilizumab-treated cases [20]. Another study showed that an adequate response to anti-TNF therapy was independently associated with both the plasma IL-23 cytokine group (Odds ratio [OR] = 0.17, 95% confidence interval [CI] = 0.04-0.73) and the IL-17A+Interferon γ+ ex-Th17 group (OR = 1.64, 95% CI = 1.06-2.54) [21]. A study conducted on asthmatics revealed that Th-17 lymphocytes and associated cytokines play a role in the mechanism of steroid hyporesponsiveness, suggesting that steroids may be less effective when IL-17 and IL-23 cytokine levels are high [22]. In our case, the use of cDMARDs, steroids, or biologics did not show a significant difference in IL-17 and IL-23 levels. A prospective study is an excellent way to understand the trend of cytokines and the impact of drugs on cytokines.

Disease activity and cytokines

The question of a pathological association between cytokines and disease severity remains unanswered despite extensive studies. We found that the majority of cases exhibited high disease activity (57.1% severe and 25% moderate). This may be due to inadequate titration of drugs, non-adherence to medication, financial issues, and limited access to healthcare. Various studies have revealed diverse results regarding the association of cytokine levels with disease activity.

In a study by Abu et al., active RA patients had significantly higher serum levels of IL-23 compared to those in remission and the control group. IL-23 also showed a positive correlation with disease activity [23]. Roşu et al. found that both serum and synovial fluid IL-17A levels had a strong positive correlation with the DAS28 score in early RA [24]. In a study by Metawi et al., both serum and synovial IL-17A levels correlated positively with DAS-28 scores (r = 0.556, p = 0.001; r = 0.392, p = 0.032, respectively) [15]. Guo et al. observed a positive correlation between serum IL-23 levels and IL-17, C-reactive protein (CRP) levels, and DAS28 in 59 RA patients [14]. In another study by Dalila et al., IL-23 levels were higher in patients with moderate to high disease activity group in 45 RA patients (p = 0.008, OR = 1.073, 95% CI = 1.019-1.130) [25]. Alsheikh et al. showed significant correlations between IL-23 levels and the DAS28 (r = 0.35, p = 0.02), CRP (r = 0.39, p = 0.02), and ESR (r = 0.45, p = 0.004) [16]. In contrast, in another study of 123 RA patients by Yamada et al., the Th17 cell level was not increased in RA and did not correlate with DAS28 [26].

In our study, both IL-17 and IL-23 levels did not differ across the severity of the disease categories (p = 0.130 and p = 0.215, respectively). Additionally, IL-17 and IL-23 showed no correlation with disease activity (rs = 0.183, p = 0.097; rs = 0.125, p = 0.259, respectively). The lack of association could be attributed to factors such as a low sample size, the use of disease-modifying antirheumatic drugs (DMARDs), and steroid use.

Functional status and cytokines

Many valid scores have been used to assess the functional status of patients in research studies. Among them, HAQ-II is an easy-to-use questionnaire-based score that can be employed in a busy clinic. The HAQ-II is a 10-item questionnaire that is as valid as the Health Assessment Questionnaire (HAQ). It is simple to use in the clinic and can be converted from HAQ to HAQ-II and vice versa [27]. In a study by Dalila et al. involving 45 RA patients, significantly high IL-23 levels were found in patients with higher grades of functional disability (p = 0.008) [25]. Metawi et al. revealed that class III functional status had higher serum IL-17A levels (17.53 ± 13.43 pg/mL) than classes I and II (8.97 ± 6.97 pg/mL, p = 0.009) [15]. In this study, only IL-23 showed a positive correlation with the functional status score (HAQ-II; p = 0.009). Therefore, a high level of IL-23 suggests a poorer functional outcome.

IL-17 and IL-23

Both IL-17 and IL-23 are closely correlated as proven by previous research. However, one cytokine may not follow the trend of the other cytokine due to various factors, probably interference from cytokines or chemokines. In a study by Stamp et al. in RA, IL-17A was observed in 13 out of 25 patients with synovia, whereas IL-23p19 was found in 23 out of 25 patients. IL-17A+ synovia showed higher expression of IL-23p19 than IL-17A- synovia (p < 0.05). IL-17 may act as an amplifier for IL-23 [28]. It was observed that IL-23 might not be present in sufficient quantity in the inflamed tissue but may have an important role in the pathogenesis of late RA [28]. In our study, IL-17 levels showed a significant positive correlation with IL-23 levels. This linear relationship suggests that the IL-17/IL-23 axis is activated to some extent in RA and might play a role in disease progression.

Limitations

Due to the low sample size of the study, correlations among many factors may not accurately represent the data for the larger population. The lack of a control group in the study limits its usefulness in predicting cytokine levels with disease activity levels. Additionally, there are many confounding factors that interfere with the analysis of cytokines in relation to various disease parameters.

## Conclusions

In our study, we observed that females exhibited higher levels of IL-23 compared to males. Serum IL-17 levels were elevated in patients with advanced (RA) when compared to those with early-stage RA. Notably, only IL-23 showed a direct correlation with CRP levels and functional status. Conversely, both IL-17 and IL-23 did not demonstrate significant correlations with disease activity, and there was no discernible difference in the cytokine levels among severity classes. Furthermore, the use of cDMARDs, steroids, or biologics did not exert a significant impact on cytokine levels.

Our findings suggest that while there may not be a direct association between cytokine levels and disease activity, elevated levels of IL-17 may potentially trigger the transition from acute synovitis to chronic, persistent arthritis and erosions leading to subsequent deformities. Additionally, a high IL-23 level may be implicated in poor functional outcomes. Therefore, early intervention aimed at blocking IL-23 and IL-17 may mitigate IL-17 activity, potentially slowing the progression to chronic inflammatory arthritis and advanced disease.

## References

[1] Scott DL, Wolfe F, Huizinga TW (2010). Rheumatoid arthritis. Lancet.

[2] Handa R, Rao UR, Lewis JF, Rambhad G, Shiff S, Ghia CJ (2016). Literature review of rheumatoid arthritis in India. Int J Rheum Dis.

[3] Niu X, Chen G (2014). Clinical biomarkers and pathogenic-related cytokines in rheumatoid arthritis. J Immunol Res.

[4] Kondo N, Kuroda T, Kobayashi D (2021). Cytokine networks in the pathogenesis of rheumatoid arthritis. Int J Mol Sci.

[5] Lubberts E (2015). The IL-23-IL-17 axis in inflammatory arthritis. Nat Rev Rheumatol.

[6] Schinocca C, Rizzo C, Fasano S, Grasso G, La Barbera L, Ciccia F, Guggino G (2021). Role of the IL-23/IL-17 pathway in rheumatic diseases: an overview. Front Immunol.

[7] Penatti A, Facciotti F, De Matteis R (2017). Differences in serum and synovial CD4+ T cells and cytokine profiles to stratify patients with inflammatory osteoarthritis and rheumatoid arthritis. Arthritis Res Ther.

[8] Lubberts E, Schwarzenberger P, Huang W, Schurr JR, Peschon JJ, van den Berg WB, Kolls JK (2005). Requirement of IL-17 receptor signaling in radiation-resistant cells in the joint for full progression of destructive synovitis. J Immunol.

[9] Raza K, Scheel-Toellner D, Lee CY (2006). Synovial fluid leukocyte apoptosis is inhibited in patients with very early rheumatoid arthritis. Arthritis Res Ther.

[10] Lubberts E (2008). IL-17/Th17 targeting: on the road to prevent chronic destructive arthritis?. Cytokine.

[11] Lubberts E, Joosten LA, Oppers B (2001). IL-1-independent role of IL-17 in synovial inflammation and joint destruction during collagen-induced arthritis. J Immunol.

[12] Rasmussen TK, Andersen T, Hvid M (2010). Increased interleukin 21 (IL-21) and IL-23 are associated with increased disease activity and with radiographic status in patients with early rheumatoid arthritis. J Rheumatol.

[13] Nakae S, Saijo S, Horai R, Sudo K, Mori S, Iwakura Y (2003). IL-17 production from activated T cells is required for the spontaneous development of destructive arthritis in mice deficient in IL-1 receptor antagonist. Proc Natl Acad Sci U S A.

[14] Guo YY, Wang NZ, Zhao S, Hou LX, Xu YB, Zhang N (2013). Increased interleukin-23 is associated with increased disease activity in patients with rheumatoid arthritis. Chin Med J (Engl).

[15] Metawi SA, Abbas D, Kamal MM, Ibrahim MK (2011). Serum and synovial fluid levels of interleukin-17 in correlation with disease activity in patients with RA. Clin Rheumatol.

[16] Alsheikh MM, El-Shafey AM, Gawish HH, El-Desoky ET (2019). Serum interleukin-23 level in rheumatoid arthritis patients: relation to disease activity and severity. Egypt Rheumatol.

[17] Combe B (2009). Progression in early rheumatoid arthritis. Best Pract Res Clin Rheumatol.

[18] Rein P, Mueller RB (2017). Treatment with biologicals in rheumatoid arthritis: an overview. Rheumatol Ther.

[19] Kageyama Y, Kobayashi H, Kato N (2009). Infliximab treatment reduces the serum levels of interleukin-23 in patients with rheumatoid arthritis. Mod Rheumatol.

[20] Lee SJ, Park W, Park SH (2015). Low baseline interleukin-17A levels are associated with better treatment response at 12 weeks to tocilizumab therapy in rheumatoid arthritis patients. J Immunol Res.

[21] Millier MJ, Fanning NC, Frampton C, Stamp LK, Hessian PA (2022). Plasma interleukin-23 and circulating IL-17A(+)IFNγ(+) ex-Th17 cells predict opposing outcomes of anti-TNF therapy in rheumatoid arthritis. Arthritis Res Ther.

[22] Vazquez-Tello A, Halwani R, Hamid Q, Al-Muhsen S (2013). Glucocorticoid receptor-beta up-regulation and steroid resistance induction by IL-17 and IL-23 cytokine stimulation in peripheral mononuclear cells. J Clin Immunol.

[23] Abu Al Fadl EM, Fattouh M, Allam AA (2013). High IL-23 level is a marker of disease activity in rheumatoid arthritis. Egypt J Immunol.

[24] Roşu A, Mărgăritescu C, Stepan A, Muşetescu A, Ene M (2012). IL-17 patterns in synovium, serum and synovial fluid from treatment-naïve, early rheumatoid arthritis patients. Rom J Morphol Embryol.

[25] Dalila AS, Said MSM, Shaharir SS, Asrul AW, Low SF, Shamsul AS, Sakthiswary R (2014). Interleukin-23 and its correlation with disease activity, joint damage, and functional disability in rheumatoid arthritis. Kaohsiung J Med Sci.

[26] Yamada H, Nakashima Y, Okazaki K (2008). Th1 but not Th17 cells predominate in the joints of patients with rheumatoid arthritis. Ann Rheum Dis.

[27] Wolfe F, Michaud K, Pincus T (2004). Development and validation of the health assessment questionnaire II: a revised version of the health assessment questionnaire. Arthritis Rheum.

[28] Stamp LK, Easson A, Pettersson L, Highton J, Hessian PA (2009). Monocyte derived interleukin (IL)-23 is an important determinant of synovial IL-17A expression in rheumatoid arthritis. J Rheumatol.

